# Metastasis: new perspectives on an old problem

**DOI:** 10.1186/1476-4598-10-22

**Published:** 2011-02-22

**Authors:** Sandra Kraljevic Pavelic, Mirela Sedic, Hrvojka Bosnjak, Sime Spaventi, Kresimir Pavelic

**Affiliations:** 1 Department of Biotechnology, University of Rijeka, 51000 Rijeka, Croatia; 2 Division of Molecular Medicine, Rudjer Boskovic Institute, 10000 Zagreb, Croatia; 3 Department of Internal Medicine, University Hospital "Sisters Of Mercy", 10 000 Zagreb, Croatia; 4 Croatian Academy of Sciences and Arts, Zrinski trg 11, 10000 Zagreb, Croatia

## Abstract

Many hypotheses have been postulated to explain the intricate nature of the metastatic process, but none of them completely accounted for the actual biological and clinical observations. Consequently, metastasis still remains an open issue with only few metastasis-inducing proteins experimentally validated so far. Recently proposed novel metastatic model, where serial and parallel metastatic processes are adequately integrated, might help to bridge the current gap between experimental results and clinical observations. In addition, the identification, isolation and molecular characterization of cancer stem cells, a population of the cells within the tumour mass able to proliferate, self-renew and induce tumorigenesis, will shed new light on the complex molecular events mediating metastasis, invasion and resistance to therapy. Understanding the molecular basis of these tumour characteristics will usher in a new age of individualized cancer therapy. In this review article, we will provide a current overview of molecular mechanisms underpinning metastasis, and discuss recent findings in this field obtained by global molecular profiling strategies such as proteomics.

## Introduction

Continuous technological advances in molecular biology have paved the way for new discoveries in cancer research. In particular, high-throughput profiling of cancer tissue specimens and body fluids has been extensively used in order to unveil specific molecular fingerprint of cancer [1-4]. Such strategy holds great promise for diagnostics purposes, as it might distinguish between different patients' prognostic subgroups (good/poor), which could provide the foundation for an individual therapeutic approach towards each patient (tailored therapy). However, in spite of these enormous efforts to elucidate cellular and molecular mechanisms underlying tumorigenesis, cancer still represents one of the deadliest scourges of the modern world.

Poor outcomes of current therapies, in particular poor prognosis for patients in advanced stages of solid tumours, have opened the possibility that tumour cells include a population of cells responsible for the initiation of tumour development, growth and its ability to metastasize and reoccur. Because these cells share some similarities with stem cells, they are referred to as cancer stem cells (CSCs). CSC are undifferentiated cells characterised by three major features: (1) potential to differentiate into several or all types of cells that are produced by the original tumour; (2) self - renewal ability; and (3) capacity to maintain the 'stem cell pool' and the most mature tumour elements for unlimited time periods [5]. CSC could originate from tissue-specific stem cells and bone marrow stem cells, and somatic cells that undergo trans-differentiation processes, or can result from the fusion or horizontal gene-transfer processes. The self-renewal and differentiation ability of CSC gives rise to all tumour cell types, and thereby produces tumour heterogeneity. This relatively new perspective, the so-called "cancer stem cell" concept, casts new light on the origins of cancer.

The relationship and differences between normal and malignant stem cells remain unclear. In many instances, normal stem cells, tumour stem cells and metastatic stem cells share some common traits. Neoplastic stem cells were indeed shown to express similar antigen pattern and to display similar functional properties in comparison with normal stem cells. Moreover, it has been shown that for the maintenance and activation of both, normal stem cells and tumour stem cells, the Wnt/beta-catenin signalling, Notch and PTEN pathways are crucial [6]. Furthermore, growth of both, normal and neoplastic stem cells, is often mediated by the same cytokines [7].

Importantly, cancer/metastatic stem cells might be discerned from embryonic stem cells by their propensity to differentiate into the cell types within a particular organ (tumour). Therefore, it is tempting to believe that tumour arises from tissue stem cells, and that cellular components bearing stem-like properties govern tumour formation. If cancer arises from rare population of cells with stem-like characteristics, then it is plausible to presume that these stem cells differ from "normal" stem cells in high rate of mutations. It is widely accepted that stem cells undergo multiple mutations that are also required for carcinogenesis, most probably due to their long-lived nature [8]. Deregulation of self-renewal mechanisms (*e.g. *Wnt/beta-catenin, Notch and Hedgehog signalling pathways), which drive the stem cell expansion, might be the early key event precipitating the formation of CSCs in the particular tissue during the onset of carcinogenesis. This hypothesis is further corroborated by the fact that oncogenes may affect different stem cells and progenitor cells resulting in phenotypic differences in tumours, whereby it was shown that transgenes encoding components of the Wnt/beta-catenin signalling pathway preferentially induce mammary cancers from progenitor cells [9]. Activation of oncogenes and inactivation of some tumour-suppressor genes as the consequence of genomic instability might drive transformation of normal stem cells to CSCs. Several genes including AKT, TRAIL and CXCL12 are recognised as candidate genes for cancer stem cell progression and latent metastasis [10]. At last, cancer/metastatic stem cells might exhibit higher expression levels of some genes (*e.g. *CXCR4, SDF1, VEGF), anti-apoptotic proteins (Bcl-2 family inhibitors of apoptosis) and transporter proteins (BCRP and P-glycoprotein), and might remain in the G_0 _phase accounting for their resistance to chemotherapy.

Methods that unequivocally identify CSC *via *specific cell-surface protein markers might be diverse but have many pitfalls. *In vivo *assays using NOD/SCID mice are expensive and time consuming. Therefore, *in vitro *long-term growth assays including sphere-formation assays, serial colony-forming unit assays and label-retention assays are often used in order to screen for stem cell fractions or CSC-regulating compounds [6]. However, the major drawbacks of these methods include: (1) lack of tissue and tumour specificity; (2) inability to isolate CSCs according to the degree of tumour differentiation; (3) lack of species-specificity of CSC receptors and their ligands and homing receptors in the tissue environment; and (4) stem cell plasticity. At last, the general problem for all *in vitro *studies is the selection pressure upon the cultured cells, resulting in the selection of certain cell population permissible to survive and proliferate under specific conditions. Isolation of CSCs is also hampered by the lack of specific CSC antigens or typical antigen combinations not identified so far. These antigens might include regular stem cells antigens such as cytokine receptors, homing receptors (integrins, selectin-ligands, chemokine receptors, cytoadhesion molecules and ligands of matrix molecules such L1 or CD44) and various drug transporters [5,6].

Metastases show a great variety of clinical presentations/manifestations, mostly in correlation with the primary tumour localization [11]. For example, breast cancer metastases can remain latent in the several years follow-up after surgical removal of the primary lesion, whereas metastases in patients with detected pancreatic cancer and small-cell lung carcinoma are often widespread at the time of cancer diagnosis. Furthermore, glioblastoma is locally progressive and invasive, but rarely affecting secondary sites outside CNS. The efficacy of common treatment regimens including radical excision of the primary tumour followed by radiotherapy and/or chemotherapy is limited and often fails to cure the patient. Recent findings imply that some treatments (*e.g. *irradiation at certain regimen, Taxol etc.) do not target the CSCs responsible for tumour development. Moreover, such treatment might have a completely opposite effect, *e.g. *to produce more cancer cells capable of metastasizing [12-14]. Indeed, it is widely accepted that CSC have a dormant nature and abundantly express drug transporters [15], which could provide a plausible explanation for resistance to standard chemotherapy known to target dividing cells. Consequently, 90% of deaths of the patients with solid tumors are attributed to local invasion and distant metastases [11].

### Metastatic model - "serial" or "parallel"?

The molecular mechanisms of metastasizing are still covered by the veil of mystery. The application of genomic profiling methods (DNA microarray) combined with either animal models of metastasis or laser capture microdissection (LCM) providing *in vivo *insight into molecular processes underlying metastatic progression have fostered the research of its complex molecular nature. So far, the so-called "serial" model of clonal progression has been generally accepted explaining that metastatic cells originate from the primary tumour and represent the end stage of tumorigenesis. Although the metastatic genotype contains additional genetic mutations, its spectrum of aberrations is thought to be similar to that found in the primary lesions [16], as augmented by the findings of kariotypic and genomic analyses of the breast, bladder, colon and kidney malignancies [17-24]. Surprisingly, some of these studies indicate the existence of metastases with lack of (without) genetic similarity to the primary tumour [18,20,25]. Bissig *et al. *[18] showed that 30% of renal cell metastases have almost completely different genotype in comparison with the primary lesion cells isolated from the same patient. Furthermore, a genomic study of metastatic breast cancer [20] presented a significant amount of breast metastases, which do not show strong clonal resemblance to the tumour of primary origin. Finally, the analysis of metastatic lesions at several sites in the same individuals showed a substantial evolutionary divergence between metastatic lesions and primary tumour, as well as between the metastases themselves.

Schmidt-Kittler *et al. *[25] reported unexpected results providing novel insight into metastatic progression. In this study, patients were divided into two groups according to clinical presence or absence of metastatic dissemination (M0- no metastatic disease; M1-metastasis positive). The results of comprehensive genomic analyses of the primary tumour specimens and single cytokeratin-positive (CK+) epithelial cells from the bone marrow of the corresponding patients were as follows: (1) the CK+ cells from M0 patients showed about half as many genomic aberrations as those from M1 patients; and (2) most of the CK+ cells from M0 patients showed little similarity to the primary tumour. To sum up, cells from M0 patients showed whole chromosome copy number aberrations, while cells from M1 patients showed sub-chromosomal changes typical for aberrations that appear during telomere crisis. The authors suggest that eventual appearance of clinically detectable metastases in M0 patients could be a consequence of early disseminated cells, which evolve and pass through crisis independently of the primary tumour. Due to slow evolution of these disseminated cells, as well as their persistence during a long period, the results of this study offer an explanation for the appearance of clinically evident metastatic disease years after treatment of the primary tumour that was presumed to be successful and curative [16]. A comparison of genetic fingerprints among disseminated cells from M0 patients with those from metastatic cells that will arise during the follow-up after primary tumour treatment is of utmost importance for the "parallel" theory. Similarly, Hüsemann *et al. *[26] showed that cancer cells can spread systematically from earliest epithelial alterations in HER-2 and PyMT transgenic mice and from ductal carcinoma *in situ *(DCIS) in women, thus providing additional evidence that supports the novel outlook on the onset of metastatic spreading and its parallel progression (evolution) during tumorigenesis. This "parallel" concept is, nevertheless, based on several pioneering studies [27,28], the first one being the experimental documentation in human leukemias [29] that shaped the concept of 'cancer stem cells'.

**Figure 1:**
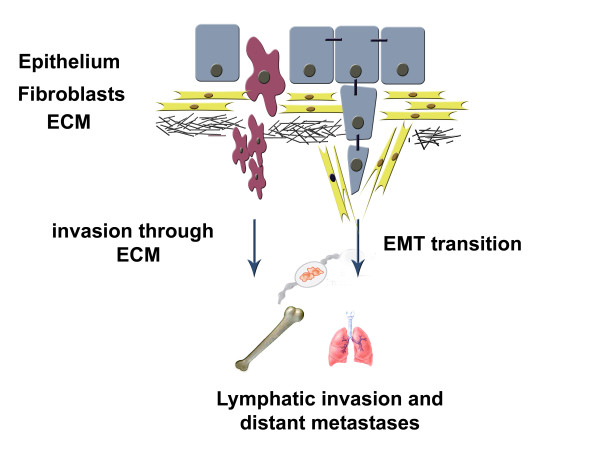
**The epithelial-mesenchymal transition (EMT) occurs at the primary tumour site where epithelial cells lose tight junctions and apico-basal polarity**. The remodelling of the cytoskeleton occurs as well. The invasion process through the extracellular matrix (ECM) is frequently led by so called tumour-associated fibroblasts. EMT can induce stem-cell-like properties in cells. The question still remains whether the existing cancer stem cells or rather those cells that escaped the primary tumour and acquired the stem cell like phenotype through the EMT process induce distant metastasis.

Since initiation of metastasis process is inherent to CSCs, the metastatic progression should be thus studied as an independent and "parallel" process in tumorigenesis governed by the so-called EMT (epithelial to mesenchymal transition) that occurs among tumour cells. The majority of tumours are epithelial but exert mesenchymal characteristics. During tumour invasion, tumour cells move from the primary tumour site and, similarly to the cells in epithelia during normal embryonic development, lose epithelial characteristics and cell-to-cell contacts, and acquire the mesenchymal gene expression [30]. Such cells are then capable of invading distant sites (Figure 1). The changes occurring in the cell-to-cell adhesions forces as well as in the cytoskeletal cortex association to plasma membranes are therefore central to the invasion, migration and intravasation of tumour cells. According to this, epithelial cells might be somehow "induced" to become metastatic by several factors such as chemokine CCL5 [31] and transcription factors FOXC2, Twist, Snug, Snail and ZEB1 [13,32] that were shown to drive the EMT programme. The EMT process might account for some similarities found between embryonic stem cells and the stem cell-like traits in neoplastic cells. One must, however, always have in mind that not every EMT-inducing factor discovered will necessarily elicit a stem-like profile.

As recently nicely reviewed by Hurt and Farrar [33], the question remains whether the existing CSCs or rather those cells that escaped the primary tumour and acquired the stem cell-like phenotype through the EMT process, induce distant metastases. Further research based on the use of powerful imaging methods and in line with the *in vivo *study performed by Condeelis *et al. *[34] showing the convincing images of individual cells delaminated from primary tumours, will be needed to resolve this issue. Taking all this together, new therapeutic modalities will be required to target independently evolved metastatic cells after early separation from the primary tumour, which will lay the groundwork for further improvement of therapeutic efficacy.

### Organ tropism and metastatic gene signature

The molecular basis of organ tropism, one of the main features of metastasis, is still obscure. It has been well documented that different types of cancer produce metastases at preferred secondary sites, depending on the tissue susceptibility to specific metastatic cells. Bone metastasis is often associated with breast, prostate or lung cancer, while it is rarely detectable in patients with diagnosed colorectal cancer [35]. This preferential development of macrometastases at distant secondary organs can be partially explained by the pattern of blood flow. Nevertheless, molecular interactions between metastatic cells (seeds) and stromal microenvironment (soil) have been proven to mediate efficiency of metastatic formation and its tissue specificity [35]. When it comes to the role of tumour microenvironment in tumour dissemination, early changes observed in tissue before evidence of carcinogenesis might be critical for tissue-specific metastasis. For example, the inflammatory response, matrix remodelling and increase in reactive oxygen species often precede tumour metastasis. Accordingly, it has been recently shown that alteration in the expression of metalloproteinase 9 precedes metastasis in lung [36]. The same studies also showed that arrival of bone marrow-derived hematopoietic progenitors expressing vascular endothelial growth factor receptor 1 in distant sites, which fosters inflammation and sustains tumour growth, represents some of the early changes present in the local microenvironment and necessary for metastases to occur [37].

Genetic and phenotypic characteristics of tumours have also been studied as factors linked to organ-specific metastases [38-40]. Kakiuchi *et al. *[39] reported differential gene expression profiles for lung, liver, kidney and bone metastases. Another research group [38] investigated metastatic potential of human breast cancer using MDA-MB-231 cell line, originally derived from the pleural effusion of a patient with metastatic dissemination. After injection into immunodeficient mice, metastases were detected in bone and adrenal medulla. Subsequently, human cells were re-isolated from the osteolytic bone metastases, and sublines with high metastatic potential were observed. Microarray profiling of these sublines revealed that the gene expression signatures directly correlated with metastases. The authors were able to distinguish cells with tendency to disseminate to bone or adrenal medulla, as the two groups showed differential gene expression patterns. A set of CXCR4, IL-11, CTGF and MMP1 genes, if co-expressed with the gene encoding osteopontin, correlated with bone-specific metastatic potential. Each of these genes, when expressed alone, failed to cause high metastatic potential. Thus, the set of genes found in the bone metastasis signature seems to be a causative factor in metastasizing to the bone. Furthermore, Minn *et al. *[40] identified a set of 54 genes with differential expression in lung-tropic breast cancer sublines in comparison with bone-tropic lines. The identified set of 54 genes in lung-tropic gene signature is probably not the only gene set responsible for metastasizing to the lungs, because a small amount of investigated primary tumours expressed this specific signature. Therefore, it is logical to presume that other lung-tropic metastatic signatures are yet to be discovered. Recent gene expression profiling studies revealed an association between Src pathway activity and late-onset bone metastasis in breast cancer, which is independent of hormone receptor status and breast cancer subtype [10]. Src activity was shown to be required for CXCL12 activation of the AKT cell survival pathway and for the resistance of metastatic breast cancer cells to the pro-apoptotic effects of TRAIL, both of which are predominantly expressed in the bone metastasis microenvironment. In view of these findings, targeting Src signalling pathway might provide a novel strategy to suppress the survival of disseminated cancer cells.

Although it is widely accepted that some primary tumours are predestined to metastasize to specific organ, it is still unclear when and how exactly they acquire organ-tropic gene signatures [41]. Furthermore, the complex multistep nature of metastasis process suggests that vast arrays of genes are responsible for its regulation. Addressing the question of metastatic prediction, recent analyses of human cancer specimens using DNA microarray technology suggest that patients can be divided into prognostic subgroups, based on the "good" or "poor" gene expression signature of the primary tumour, which predicts the risk of metastasis appearance after tumour resection, particularly evaluated in breast cancer [4,42,43]. Traditionally, in clinical practice, the potential of its metastatic recurrence is in correlation with primary tumour size and histological grade. In a recent study, Ramaswamy *et al. *[44] compared gene signatures of primary adenocarcinomas and metastases from a similar set of adenocarcinomas. Their analysis revelaed a "metastatic gene signature" common to many different tumour types. When detected in some primary tumours, this signature indicated its pre-existed tendency to metastasize and, therefore, had a significant prognostic value. Nevertheless, evaluation of this metastatic signature once the primary tumour is diagnosed distinguishes tumours with local growing potential from those preconfigured to disseminate to distant sites [44,45]. However, it remains unclear whether these metastatic signatures are causative factors in metastatic spreading or indirect indicators of metastatic potential [46].

Besides screening for genes linked to metastasis organ tropism, the stem cell theory might provide an additional explanation for this phenomenon. In fact, it has been well documented that CSCs express a G-protein-coupled seven-span transmembrane receptor CXCR4 on their surface similarly to normal stem cells for different organs/tissues [47]. Facts about the role of chemokines, small pro-inflammatory chemoattractant cytokines that bind to G-protein transmembrane receptors of target cells, in mediating cell trafficking might additionally illuminate how metastases are attracted to specific organs. For example, a stromal-derived chemokine SDF-1 exclusively binds to CXCR4 and is highly expressed in lymph nodes, lung, liver or bones. Therefore, metastasis of CXCR4+ tumour cells might be driven specifically to these organs through the SDF1-CXCR4 axis [47]. This assumption is augmented by the findings revealing that several CXCR4+ cancers such as breast, ovarian and prostate cancer metastasize to bones from bloodstream in a SDF-1 dependent fashion [47,48]. However, possible therapeutic strategies based on modulation of the SDF1-CXCR4 axis or other chemokines involved in stem cell trafficking should be carefully considered, as this signalling pathway is normally involved in the trafficking of stem cells.

### Molecular mechanisms of metastasizing

Metastasis is apparently central in terms of clinical management of cancer, as the preponderance of patients' deaths are associated with disseminated disease rather than the primary tumour. Moreover, patients with small primary tumours and node negative status (T1N0) at surgery often (15% to 25%) develop distant metastases [49]. As summarized in this paper, recent literature data support the concept of metastasis as a second disease imposed on the primary tumour, where the outcome of metastasis is determined by the interplay between the specific subpopulation of metastatic cells and host homeostatic factors in specific organ microenvironment including vasculature. However, over the past decades, the so-called progression model has been widely accepted among clinicians and researchers. This model depicted metastasis as a result of several consecutive mutational events occurring either in subpopulations of the primary tumour or disseminated cells, and yielding a small fraction of cells that acquire full metastatic potential. Recently, several microarray studies have prompted reexamination of the progression model, as they enable identification of gene signatures that can distinguish metastatic from non-metastatic tumours, and postulate that metastatic propensity is established early in oncogenesis [44,45]. Accordingly, metastatic genes can be classified into several groups: metastasis initiation genes, metastasis progression genes and metastasis-virulence genes [11]. Metastasis initiation genes provide an advantage in a primary tumour and enable tumour cells to enter the blood-flow. Most genes mediating tumour cell motility, invasion or angiogenesis belong to this group. This class includes genes involved in EMT and caspase 8, whereby loss of caspase 8 function protects tumour cells from programmed death due to release of integrin-regulated anchoring at the invasive front [30,50,51]. The second class of metastatic genes, specified as the metastasis progression genes fulfil some rate-limiting functions in primary tumour growth and other specific functions in metastatic colonization. These genes can be found within organ-tropic gene signatures, thus causing specific advantage restricted to particular distant organ. Nevertheless, while metastasis-virulence genes participate in metastatic colonization due to selective advantage in secondary sites, they do not affect the primary tumour development [11]. Therefore, these genes promote aggressiveness of metastatic tumour cells at the level of colonized distant organs. Recent study dealing with organ-specific metastases in breast cancer reported on the lung-tropic metastatic gene set comprising 54 genes, among which only 18 were expressed in primary tumours. These results provide an evidence for the existence of metastasis progression genes and metastasis virulence genes [40,52]. Both groups of genes enable circulating tumour cells to colonize the lungs. The 18 genes expressed in the primary tumour induce primary tumorigenesis, leading to the larger tumour size at the time of diagnosis. Due to vascular-remodelling programme, three out of those 18 genes (EREG, COX2 and MMP1) facilitate tumour angiogenesis and intravasation in mammary tumours. In addition, they act as a mediator in tumour cells extravasation from the lung capillaries. Accordingly, the EREG, COX2 and MMP1 genes are classified as metastasis progression genes.

Introduction of improved molecular research methods has opened new chapter in metastasis research by specifically disclosing some of the underlying molecular events in metastatic progression. Molecules which play an important role in processes involving cell-cell adhesion, migration, proteolysis, chemotaxis, angiogenesis and signal transduction have been investigated with aim to decipher their activity in evolution of metastasis [35]. Five candidate metastasis genes, namely CXCR4, IL-11, CTGF, MMP1 and osteopontin from the bone metastasis signature reported by Kang *et al *[38] encode secreted cytokines or cell surface receptors, which is consistent with traditionally accepted idea that metastasis formation is a consequence of pathological interactions between tumour cells and stromal microenvironment. The evidence for this widely accepted theory is provided by Hu *et al. *[53], who used a xenograft model of human DCIS and primary human breast tumours. The authors found that myoepithelial cells and fibroblasts mediate the transition from DCIS to invasive carcinoma. In the absence of normal myoepithelial cells, co-injection of fibroblasts promoted progression of *in situ *to invasive carcinoma. On the contrary, co-injection of normal myoepithelial cells effectively suppressed tumour weight, despite presence of progression-promoting fibroblasts. The obtained results were not the upshot of permanent genetic aberrations in the epithelial tumour cells. Furthermore, TGF-β and Hedgehog signalling were recognized to have a critical role in breast tumour progression, while decreasing TGF-β and Hedgehog pathway activity *via *TGFBR2/SMAD4 downregulation and Gli2 expression resulted in the loss of myoepithelial cells and accelerated invasion.

Even if a wealth of evidence supports this model, some paradoxes still remain. First, in many tissues where tumours arise, normal mature cells have a short lifespan. Consequently, the chances to accumulate mutations required for tumour development are rather limited. Secondly, patients diagnosed with disseminated disease with unknown primary cancer metastatic disease have no clinically detectable primary tumour or only a small, well differentiated lesion that is found at autopsy [54]. Furthermore, although variant clones with high metastatic capacity can be identified in populations, it is frequently observed that these variants revert to a low-metastatic capacity after several generations [55,56]. The so-called transient metastatic compartment model proposed by Weiss *et al *[57] suggests that all viable cells in a tumour might acquire metastatic capacity as explained by the progression model, but due to their position in the primary tumour and random epigenetic events, only a small fraction of these cells are competent to metastasize at a given moment in time. Thus, not all cells within a tumour preserve the capacity to disseminate to secondary sites as a result of accidental, or microenviromentally induced epigenetic events or inadequate access to vasculature. However, this model fails to explain the clonal nature of metastases. If every cell had a metastatic ability modulated only by momentary epigenetic events, then it would be less likely that significant proportions of secondary tumours would appear to be of clonal origin.

Another intriguing theory, the so-called cell fusion theory, explains that the acquisition of metastatic phenotype occurs when a healthy migratory leukocyte fuses with a primary tumour cell. Such a 'hybrid' has the innate blood cell ability to migrate through the body while still keeping the uncontrolled cell processes as occurring in the tumour cells [58,59]. Interestingly, the fusion of genetic and cytoplasmic material between cells of different origins is an important physiological process during development. Generally, cell fusion and horizontal gene-transfer events could be important in the development of the CSCs [60]. Substantiating these theses, the i*n vitro *fusion of cells was proved to produce subclones with varying metastatic potentials [61,62], and over 30 reports confirming cancer cell fusion in animal tumour models have been published so far [63,64]. In addition, a number of factors such as cellular or viral fusion proteins or environmental factors may provoke cell fusion of tumour and normal cells. Such fused cells will probably die or become quiescent. Nevertheless, there is a small fraction that will maintain the ability to proliferate and generate malignant cells. It has been shown that endothelial cells in solid tumours might be aneuploid with multiple chromosomes and multiple centrosomes, implying the possibility of cell fusion between tumour cells and endothelial cells [65]. This theory has yet to be confirmed in humans as well.

### Technological advances in metastasis research: proteomics

Global transcriptome profiling of cancer has undoubtedly improved our knowledge of tumour biology, and has led to the discovery of novel therapeutic and imaging targets, as well as potential prognostic and predictive biomarkers for diverse cancer types. However, transcriptomics can predict neither the expression level nor the functional status determined by folding, post-translational modifications, cellular localization and molecular interactions of the key signalling molecules in complex protein networks integral to cancer pathogenesis. For example, how exactly and when the EMT process and other pro-survival signalling cascades in cancer cells are triggered, still remains to be elucidated. Cancer may be genetically based, but on the functional level, it is a proteomic disease [66], because tumour progression, invasion, and metastasis depend on the functional activity of many proteins, such as growth factors and proteases.- Consequently, molecular oncologists have been turning more to proteomics technologies (Table 1) as to identify novel protein biomarkers specifically associated with metastatic transition, and to decipher signal transduction pathways that propel the cells down the road towards metastasis. Such technological approach should provide an early detection and prediction of metastatic processes, and reveal novel targets for drug development and therapeutic intervention. There is now an enormous wealth of literature data on employing proteomics in metastasis research covering practically all tumour types, and discovered biomarkers might be correlated with the metastatic process and/or cancer stem cell phenotype. Some recent examples will be briefly discussed below.

**Table 1:** Overview of the most common proteomics technologies in the research of tumour invasion and metastasis

Proteomics method	Abbreviation	Basic principle	Biological Application	Advantages	Limitations
Two-dimensional gel electrophoresis	2-DE	Proteins are first resolved by their isoelectric points, and then by molecular weights	Separation of proteins in complex biological samples	High resolution Very sensitive Direct detection of post-translational modifications	Limited automation Problematic gel-to-gel reproducibility Problematic recovery of hydrophobic and large molecular weight proteins Limited dynamic range of detection
Two-dimensional difference gel electrophoresis	2D-DIGE	Samples are labelled with two spectrally distinct fluorescent cyanine dyes, and run on the same 2-DE gel; the two gel images corresponding to each dye scan are then overlaid, and the intensities of paired spots are compared across the gel images	Quantification of the differences in protein expression between different samples	High sensitivity Accurate quantitation Good reproducibility	Expensive fluorophores, equipment and software
Matrix assisted laser desorption ionisation time-of-flight mass spectrometry	MALDI-TOF MS	Tryptic digests of sample proteins are co-crystallized with matrix, and spotted onto MALDI plate; ionization occurs by pulsed laser radiation primarily absorbed by the matrix, causing desorption and ionization of the analyte; the resulting peptide ions are directed into TOF mass analyzer, where peptide masses are measured by determining the time required for the ions to traverse the length of the flight tube and reach detector	Protein identification Amino-acid sequencing Determination of the type and position of post-translational modifications	Produces less raw data than other MS techniques Data are relatively easy to interpret since most peptides carry only one charge and are present as a single peak in a spectrum	Requires previous separation of protein mixture Hampered identification of small acidic and integral membrane proteins
Multidimensional protein identification technology	MudPIT	Mixture of tryptic peptides is resolved by the microcapillary column packed with reversed-phase resin followed by strong cation exchange resin; peptides are eluted directly from the column into the mass spectrometer to be rapidly analyzed	Large-scale protein analysis of complex biological mixtures Identification of protein complexes Determination of post-translational modifications Quantitative analysis of protein expression	Detects proteins of wide range of pI, abundance and sub-cellular distribution Employed directly on crude samples Easily automated High resolving power High sensitivity	Time-consuming Requires experienced personnel Does not detect protein activity nor interactions Limited throughput Generates the vast stream of raw data
Surface enhanced laser desorption and ionization time-of-flight mass spectrometry (ProteinChip Technology)	SELDI-TOF MS	Protein solutions are applied to the spots of ProteinChip Arrays that contain either chemically (anionic, cationic, hydrophobic, hydrophilic, or metal ion) or biochemically (immobilized antibody, receptor, DNA, enzyme, etc.) active surface retaining proteins according to their specific physicochemical properties; after adding matrix solution to bound proteins, the latter are ionized with nitrogen laser and their molecular masses measured by TOF mass analyzer. As a result, unique protein abundance profiles of species bound to the chip surface are obtained.	Biomarker discovery Characterization of protein-protein and protein-DNA interactions and post-translational modifications (glycosylation and phosphorylation)	Suitable for crude biological samples (body fluids, cells) High-throughput capability High sensitivity Detects proteins with molecular weights lower than 6-kDa High precision and reproducibility	Additional MS analysis needed for determining the identity of differentially expressed protein species
Isotope-coded affinity tags	ICAT	Two different protein samples are labelled at cysteines with the isotopically light and heavy ICAT reagents, combined and digested with trypsin; ICAT-labeled peptides are isolated by avidin affinity chromatography and analyzed by HPLC coupled to a tandem mass spectrometer; the ratio of ion intensities from co-eluting ICAT-labeled pairs permits the quantification, while a subsequent MS/MS scan provides the protein identification	Sequence identification and quantification of proteins in complex mixtures Analysis of protein changes in specific subcellular fractions	Selects only cysteine-containing peptides and thus effectively reduces the complexity of the peptide mixtures	Incomplete proteome coverage (10-20% of the whole cell proteome)
Laser-capture microdisscetion	LCM	A stained tissue slide is placed under a microscope, and a specific thermoplastic polymer film is placed over the tissue; the cells of interest are shot by an infrared laser pulse, which melts and fuses the film around the targeted cells; the cells embedded in the polymer are lifted away from the remaining tissue	Isolation of pure cell populations from heterogeneous tissue sections prior to proteomic analyses focused on the investigation of novel biomarkers and drug targets	High-throughput Reduces sample heterogeneity Increases the specificity of signals obtained in downstream protein analysis	Requires competency in identifying the cells of interest Limited timeframe for microdissecting fresh frozen tissue.
Reverse-phase protein microarrays	RPMA	Cell lysates are arrayed on nitrocellulose-coated glass slides binding denatured proteins; the slide is probed with a single antibody specific for an antigen of interest; upon signal development and imaging, the relative proportion of the analyte protein molecules can be compared between test samples on the array	Functional mapping of known cell-signalling networks or pathways Characterization of protein-protein, protein-DNA, and/or protein-RNA interactions	High-throughput Requires low sample volume Extremely sensitive analyte detection Good reproducibility, sensitivity, and robustness	The lack of availability of high-quality, specific antibodies Hampered analysis of low-abundance post-translational events

2-DE followed by MALDI-TOF MS analysis represents the workhorse of the vast majority of published proteomics studies on metastasis research, and this approach has proved effective in measuring protein expression patterns within cells, tissues and bodily fluids uncovering many novel metastasis-related proteins, such as chloride intracellular channel 1 (CLIC1). CLIC1 was specifically correlated with metastasis of gallbladder carcinoma, the most frequent form of bile duct cancer, as its expression was significantly up-regulated in the highly metastatic gallbladder cancer GBC-SD18H cell line when compared to the poorly metastatic GBC-SD18L cell line [67]. In addition, the overexpression of CLIC1 promoted cell motility and invasion of GBC-SD18L cells, while RNA interference of CLIC1 remarkably decreased cell motility and invasive potency of GBC-SD18H cell line. Similarly, proteomics profiling of tumor tissues from gastric cancer revealed CLIC1 to be significantly up-regulated in 67.9% of the patients [68]. This study revealed significant correlation between elevated expression of CLIC1 and lymph node metastasis, lymphatic invasion, perineural invasion, advanced pathological stage and poor survival in gastric cancer, which highlights the role of CLIC1 in tumor invasion and metastasis in gastric cancer.

S100A11 protein is a calcium-binding protein implicated in a variety of biological functions such as proliferation and differentiation, whose relation with tumor progression and invasion was substantiated by recent proteomics studies. Tian *et al. *[69] carried out comparative 2-DE/MALDI-TOF MS analysis of non-metastatic and highly metastatic non-small cell lung cancer (NSCLC) cell lines and found S100A11 to be specifically up-regulated in the metastatic cell line. Immunohistochemical staining of 65 primary NSCLC tissues and 10 matched local positive lymph node specimens confirmed that the over-expression of S100A11 in NSCLC tissues was significantly associated with higher tumor-node-metastasis stage and positive lymph node status, implying regulatory role of this protein in promoting invasion and metastasis of NSCLC [69]. Using the same proteomics strategy, S100A11 was also reported to be up-regulated in metastatic hepatocellular carcinoma (HCC) tissues [70], and to rise in the expression level with the progression of colorectal cancer [71]. In view of these studies, S100A11 protein might be considered as useful candidate molecule for early diagnosis and intervention of NSCLC, HCC and colorectal metastases.

The development of fluorescent dye labels allowing the comparison of two different samples on a single gel referred to as 2-D fluorescence difference gel electrophoresis (2-D DIGE) has improved reproducibility, more accurate quantitation and spot statistics between gels in comparison with conventional 2-DE. This method was successfully applied to screening potential biomarkers for early detection of prostate cancer and lung squamous carcinoma metastases. Pang *et al. *[72] analyzed protein samples from localized and lymph node metastatic prostate cancer (LNM PCa) as well as benign prostatic hyperplasia tissues, and found increased expression of e-FABP5, MCCC2, PPA2, Ezrin and SLP2 along with reduced expression of SM22 in LNM PCa tissues. Importantly, e-FABP5 levels were significantly increased in the sera of patients with LNM PCa. These findings were in line with the previous studies revealing an over-expression of e-FABP5 protein in PCa tissues [73]. Overexpression of e-FABP5 was shown to induce metastasis by up-regulating VEGF, which plays a crucial role in the metastatic cascade [74]. Therefore, increased e-FABP expression is a possible target to inhibit the malignant progression of prostate cancer cells. Using the same technique, Yao *et al. *[75] compared the protein profiles between laser capture-microdissected (LCM) lung squamous carcinoma (LSC) cells with and without lymph node metastasis (LNM), and found rise in the expression level of HSP27, Annexin A2, and CK19, whereas 14-3-3 σ had reduced expression in LNM LSC. Additional immunohistochemical analyses confirmed that these proteins are indeed correlated with several clinicopathological variables and prognosis of LSC.

The increasing use of high-throughput platforms for the analysis of protein expression levels driven by technological improvements in mass spectrometry and array-based technologies has pushed the boundaries of clinical oncoproteomics. Serum protein pattern profiling by ProteinChip Technology (SELDI-TOF MS) has emerged as novel approach to discover protein signatures capable of discriminating patients with primary cancer from those with metastasis, as demonstrated by several recent studies on laryngeal squamous cell carcinoma [76], colorectal [77], ovarian [78], lung [79], prostate [80], breast [81] and gastric cancer [82]. Collectively, these studies clearly showed diagnostic and prognostic value of ProteinChip technology. In addition, Goncalves *et al. *[83] used this proteomics approach on a high-risk early breast cancer population receiving standard adjuvant chemotherapy, and managed to identify a post-operative serum proteomic profile that might predict metastatic relapse. Currently, there are no satisfactory screening and early diagnostic strategies for metastatic cancer. Due to its quantification capability and reproducibility, SELDI-TOF MS represents a serious candidate tool for rapid and accurate high-throughput screening of cancer patients.

Major breakthrough in metastasis research represents the combination of LCM and protein microarray technologies, which has been applied to the analysis of human metastatic breast and ovarian cancer tissue samples in phase II clinical trials at the National Institutes of Health National Cancer Institute [84]. Similarly, Sheehan *et al. *[85] utilized reverse phase protein microarray (RPMA) technology to profile a matched cohort of primary and metastatic ovarian carcinomas using phosphorylation-specific antibodies. Strikingly, the metastatic signatures were clearly very different from the primary tumor taken at the same time at surgery, and these fingerprints appeared to be virtually patient-specific, which underscores the critical need for patient-tailored therapy designed to specifically target the disseminated cells. The same study revealed several phosphorylated proteins that were differently expressed between primary and metastatic tissues, including the phosphorylated forms of c-Kit, Ask, myristoylated alanine-rich C kinase substrate, IκBα, and Ras-GRF [85]. Importantly, metastasis correlated with activation of c-Kit, which was previously demonstrated to pertain to advanced stage and chemotherapy resistance in serous ovarian carcinomas.

RPMA technology has also proved beneficial in investigating the cellular events that accompany metastatic progression, as exemplified by Paweletz *et al *[86] who used RPMAs to compare LCM-specimens of histologically normal prostate epithelium, prostate intraepithelial neoplasia, and invasive prostate cancer. Amplification of antibody-antigen complexes on the microarrays revealed a statistically significant increase in phosphorylation of Akt and a decrease in phosphorylation of Erk in premalignant and invasive prostate cancer. The authors draw a conclusion that these shifts in protein abundance indicated activation of pro-survival signaling pathways with cancer invasion. The same study also demonstrated that downstream components of the apoptotic cascade, namely cleaved and noncleaved caspase-7 and PARP were also shifted towards prosurvival function during cancer progression [86]. Based on obtained data, the authors proposed a hypothetical model of prostate cancer progression according to which activation of Akt suppresses apoptosis, probably *via *inactivation of its substrate GSK3-β, which might cause an imbalance between cell proliferation and death leading to the accumulation of cells within prostate gland [86]. Simultaneously, transient ERK activation and turning on pro-survival pathways might be associated with cellular migration responsible for invasion. Akt seems to play a central role in prostate cancer metastasis, as its activation could foster cell motility and survival during stromal invasion.

Despite rapid development of proteomics technologies, cancer stem cell proteomics is still in its infancy. This could be ascribed to the extremely low availability of putative CSCs, as these cells represent only a small fraction of the overall cancer cell population. In addition, the methods for isolating a large enough sample of pure CSCs have not been developed yet [87]. However, recent proteomics studies on leukemic [88,89] and pancreatic cancer stem cells [90] are bright examples on how to successfully overcome the issue of sample limitations in CSC research. Tibes *at al. *[88] demonstrated that RPMAs could be reliable, reproducible high-throughput approach for analyzing protein expression and phosphorylation status in primary acute myelogenous leukemia cells, cell lines and stem cells. Importantly, this group of authors found that leukemic stem cells had apparently different protein signature compared with normal stem cells, and observed different levels of protein expression when normal and leukemic CD34+/CD38+ and CD34+/CD38- cells were compared, or when leukemic and normal stem cells were compared. Novel perspective on human leukemogenesis was provided by Ota *et al. *[89], who utilized 2-DE/MS for protein profiling of isolated AC133^+ ^leukemic blasts from 13 individuals with acute leukemia or related disorders. They detected 10 differentially expressed proteins including NuMA, heat shock proteins, and redox regulators. An over-expression of HSP70 family proteins in leukemic blasts was proposed to be directly associated with leukemogenesis, or to the development of drug resistance. Importantly, the finding that the abundance of the nuclear mitotic apparatus protein (NuMA) in leukemic blasts was related to the number of chromosomal abnormalities raised the possibility that over-expression of NuMA perturbs cell cycle progression by inhibiting mitosis resulting in the chromosomal instability. The finding that the forced expression of NuMA resulted in G2/M arrest and apoptosis clearly shows that some other genetic events, besides aberrant expression of NuMA, are required for malignant transformation to leukemic cells.

Characterization of the CSCs opens a new avenue for designing novel therapeutic strategies against cancer. However, crucial to this task will be identification of specific cell surface antigens (markers) (Table 2) for CSCs detection and isolation from the heterogeneous tumor population. Hereby, membrane proteomics plays an important role, as demonstrated by He *et al. *[91], who employed the combination of lectin microarray and liquid chromatography-tandem mass spectrometry (LC-MS/MS) to discover novel cell surface glycoprotein markers of a glioblastoma-derived stem-like cell line. These authors identified six differentially expressed proteins between the stem-like glioblastoma neurosphere culture and traditional adherent glioblastoma cell line, whereby receptor-type tyrosine-protein phosphatase zeta, Tenascin-C, Chondroitin sulfate proteoglycan NG2, Podocalyxin-like protein 1 and CD90 were up-regulated, and CD44 was down-regulated [91]. Further elucidation of the biological roles of these proteins might prove important for an early diagnosis and improved treatment of glioblastoma.

**Table 2:** Representative cell surface markers for human cancer stem cells

Type of cancer stem cells	Cell surface markers
Acute myelogenous leukemia;	CD34+, CD38-, CD44, CD123+
Chronic myeloid leukemia	CD34+, CD38-, CD123+
B-acute lymphogenous leukemia	CD34+, CD38-, CD19+
Ph1-acute lymphogenous leukemia	CD34+, CD38-
Blast-crisis CML	CD34+, CD38+, CD123+
Myeloproliferative disorder	CD117+
Glioblastoma	CD133+
Medulloblastoma	CD133+
Pilocytic astrocytoma	CD133+
Anaplastic ependymoma	CD133+
Breast	CD44+, CD24^-/low^, ESA+
Prostate	CD133+/alpha 2 beta 1 integrin/CD44+ CD44+/CD24-
Ovarian cancer	CD44+, MyD88+
Colon cancer	CD133+, CD44+, CD166+, E-CAMhig
Pancreatic cancer	CD133+, CD44+, CD24+
Hepatocellular cancer	CD133+
Head and neck squamous cell carcinoma	CD44+
Bone sarcomas	Stro-1+, CD105+, CD44+
Melanoma	CD20+, CD133+
Lung cancer	CD133+
Liver	CD133+, CD90+
Central nervous system	CD133+

Although yielding many useful information on cancer invasiveness and progression, conventional genomic and proteomic platforms are still limited in their capacity to identify changes in protein activity caused by post-translational mechanisms [92]. Most proteomic techniques provide information on protein abundance, which does not necessarily correlate with enzyme activity, since most enzymes are expressed as inactive zymogens or reside in complex with their endogenous inhibitors [93]. As an alternative, a new strategy termed activity-based protein profiling (ABPP) has emerged based on the use of enzyme family-specific activity-based chemical probes (ABPs) linked to specific reporter groups that by nature only target and subsequently tag the active form of these enzymes both *in vitro *and *in vivo *[94]. Jessani et al. [95] used this approach to profile serine hydrolase activities across a panel of human breast and melanoma cancer cell lines, and found that highly invasive cancer cells exerted secreted/membrane serine hydrolase activity profiles nearly orthogonal to those displayed by their less aggressive counterparts indicating that invasive cancers may share proteomic signatures that are more reflective of their cellular phenotype than tissue of origin. Importantly, they detected the up-regulation of two enzyme activities in invasive cancer lines, namely urokinase, a secreted serine protease with previously established role in tumor progression, and a membrane-associated serine hydrolase KIAA1363, the latter implicated as a new marker of tumor progression [95]. Similar study combining ABPP and metabolomics (rapid, high-throughput characterization of the small molecule metabolites including any metabolic intermediates, hormones and other components of signaling pathways found in an organism [96]) established a central role of KIAA1363 in an ether lipid signaling network bridging platelet-activating factor and lysophosphatidic acid [97]. As evident from these studies, integration of several different global profiling technologies may illuminate biochemical networks pertinent to cancer development and progression. In this respect, metabolomic profiling has already proved beneficial in characterizing the metabolic features of hepatocellular carcinoma [98], breast cancer [99], renal cell carcinoma [100] and prostate cancer [101] metastases. Metabolic alterations during cancer progression and metastasis revealed by these studies might provide new putative diagnostic and prognostic biomarkers as well as new therapeutic targets, such as e.g. sarcosine, an *N*-methyl derivative of glycine elevated most robustly in metastatic prostate cancer and detectable in the urine of men with organ-confined disease [101]. However, metabolic profiling studies involving CSCs to identify the key metabolites inherent to tumor progression are still scarce. The pioneering work in this field was performed at the American company Stemina Biomarker Discovery, whose scientists are focused on identifying metabolomic biomarkers that are potential indicators of drug efficacy against CSCs for the establishment of novel drug screening assays (http://www.stemina.com/web/publications.php). Specifically, they found small molecules unique to CSCs derived from glioblastoma multiforme (GBM), and identified unique metabolomic footprint of three different brain tumor stem cell lines (BTSC). Besides their potential as a screening tool for measuring GBM presence and progression, identified metabolite molecules might also serve as therapeutic targets in order to manufacture drugs specifically targeting GBM and BTSC cells.

## Conclusion

Many hypotheses have been postulated to explain the intricate nature of the metastatic process, but none of them completely accounted for the actual biological and clinical observations. Consequently, metastasis still remains an open issue with only few metastasis-inducing proteins experimentally validated so far. Global scale proteomics studies undoubtedly revealed specific metastatic markers often related to cell-signalling processes; however, they proved to be patient-specific rather than type- or tumor stage-specific, which necessitates a need for individual therapeutic approach towards each patient.

Due to inconsistency between experimental results and clinical observations, a novel metastatic paradigm where serial and parallel metastatic processes are adequately integrated is needed to account for these differences. In addition, organ-tropic gene signatures were shown to bear a potential to improve patient risk stratification and therapeutic treatment. New diagnostic tools are therefore urgently required in clinical practice to detect patients who will benefit from the adjuvant chemotherapy after primary tumour resection. Consequently, this will lead to the improvements in patient treatment and reduction of adverse effects in patients who are traditionally unnecessarily treated with chemotherapy. While study of the metastases molecular mechanisms is far from trivial, the results discussed in this paper suggest that the benefits to our understanding of the cellular basis of metastasis more than justify the efforts employed.

## Competing interests

The authors declare that they have no competing interests.

## Authors' contributions

SKP coordinated the preparation of the manuscript, designed the manuscript structure, performed the literature search, wrote the majority of manuscript text and prepared accompanying Figure 1 and Tables 1 and 2. MS has been involved in extensive literature search, language revision, preparation of Tables 1 and 2 and wrote the majority of the section "Technological advances in metastasis research: proteomics". HB has been involved in literature search and revision of clinical aspects in the manuscript and wrote majority of section "Organ tropism and metastatic gene signature". SS contributed to the main manuscript idea, concept preparation, literature search and revised the final manuscript. KP proposed the manuscript idea, and has been involved in literature search, manuscript writing (in particular sections dealing with concept of cancer stem cells and molecular mechanisms underlying metastases), review of clinical aspects and final manuscript review.

All the authors read and approved the final manuscript.
